# Forest-to-pasture conversion increases the diversity of the phylum *Verrucomicrobia* in Amazon rainforest soils

**DOI:** 10.3389/fmicb.2015.00779

**Published:** 2015-07-30

**Authors:** Kshitij Ranjan, Fabiana S. Paula, Rebecca C. Mueller, Ederson da C. Jesus, Karina Cenciani, Brendan J. M. Bohannan, Klaus Nüsslein, Jorge L. M. Rodrigues

**Affiliations:** ^1^Department of Biology, The University of Texas at ArlingtonArlington, TX, USA; ^2^Institute of Ecology and Evolution, University of OregonEugene, OR, USA; ^3^Embrapa AgrobiologiaSeropedica, Brazil; ^4^Centro de Energia Nuclear na Agricultura, University of Sao PauloPiracicaba, Brazil; ^5^Department of Microbiology, University of MassachusettsAmherst, MA, USA; ^6^Department of Land, Air and Water Resources, University of California, DavisDavis, CA, USA

**Keywords:** tropical forest, microbial biodiversity, land use change, habitat filtering, biotic homogenization

## Abstract

The Amazon rainforest is well known for its rich plant and animal diversity, but its bacterial diversity is virtually unexplored. Due to ongoing and widespread deforestation followed by conversion to agriculture, there is an urgent need to quantify the soil biological diversity within this tropical ecosystem. Given the abundance of the phylum *Verrucomicrobia* in soils, we targeted this group to examine its response to forest-to-pasture conversion. Both taxonomic and phylogenetic diversities were higher for pasture in comparison to primary and secondary forests. The community composition of *Verrucomicrobia* in pasture soils was significantly different from those of forests, with a 11.6% increase in the number of sequences belonging to subphylum 3 and a proportional decrease in sequences belonging to the class *Spartobacteria*. Based on 99% operational taxonomic unit identity, 40% of the sequences have not been detected in previous studies, underscoring the limited knowledge regarding the diversity of microorganisms in tropical ecosystems. The abundance of *Verrucomicrobia*, measured with quantitative PCR, was strongly correlated with soil C content (*r* = 0.80, *P* = 0.0016), indicating their importance in metabolizing plant-derived carbon compounds in soils.

## Introduction

The Amazon rainforest is the largest equatorial forest in the world, encompassing 40% of the world’s tropical ecosystems (Laurance et al., 2001). It maintains one-fifth of the world’s total freshwater volume, influencing hydrological and climatological cycles and balancing the global flux of atmospheric gasses. The Amazon rainforest harbors an estimated 40,000 vascular plant species, 5,500 vertebrate species, and 100,000 invertebrate species (Da Silva et al., 2005; Lewinsohn and Prado, 2005). The Amazon rainforest is under constant threat from agricultural conversion, with approximately 16% of the original cover already lost (Soares-Filho et al., 2006). Land use change driven by human activities is considered the most important factor for biodiversity losses in the tropics (Sala et al., 2000) and a large number of studies have documented the negative effects of land use change on plants and animals (Gibson et al., 2011; Wearn et al., 2012).

Recently, it has been shown that forest-to-pasture conversion also impacts microorganisms, resulting in biotic homogenization of communities (Rodrigues et al., 2013). Specifically, there was a substantial decrease in the abundance of *Acidobacteria* 16S rRNA gene sequences and correspondent increase in sequences identified as belonging to the phyla *Firmicutes*, *Actinobacteria*, and *Chloroflexi.* Other phyla did not show an apparent response to conversion, including the *Verrucomicrobia;* however, this group has exhibited strong responses to land use change in previous studies (Kielak et al., 2008; Suleiman et al., 2013; Montecchia et al., 2015; Prober et al., 2015) and it is possible that shifts within the *Verrucomicrobia* were masked by limited coverage within the overall bacterial community (Bergmann et al., 2011).

The *Verrucomicrobia* is a ubiquitous and abundant group in many terrestrial ecosystems (Buckley and Schmidt, 2001; Janssen, 2006; Bergmann et al., 2011; Fierer et al., 2013). The ecology of this group is unclear, but they have potential roles in methane oxidation and polysaccharide degradation (Martinez-Garcia et al., 2012), and the high relative abundance of this group in soils across a wide range of ecosystems suggests that they play important, but poorly understood, roles. Members of the *Verrucomicrobia* are difficult to isolate and, to date, only a limited number of isolates (total of 31) have been obtained from soil samples (RDP II)^1^, with four out of seven classes remaining without representatives in culture (Hedlund, 2011). The abundance and class composition of this phylum remain virtually unknown in tropical forest, and it is unclear to what extent members of this phylum can recover from agricultural conversion when pastures are abandoned and secondary forests are established.

In this study, we sought to fill the knowledge gap regarding the abundance and class-level distribution of *Verrucomicrobia* in the Amazon rainforest by addressing the following questions: (i) How is the diversity and community composition of *Verrucomicrobia* in Amazon rainforest soils affected by deforestation? and (ii) What are the environmental variables associated with the presence of *Verrucomicrobia* in Amazon rainforest soils? We hypothesized that the abundance and diversity of this phylum would be higher in the primary forest than in adjacent pasture and secondary forest sites. In order to answer the above questions, we designed and tested a specific oligonucleotide PCR primer for the detection of the phylum *Verrucomicrobia*, determined the effects of forest-to-pasture conversion on their community structure, and correlated the presence of members of *Verrucomicrobia* to local environmental conditions.

## Materials and Methods

### Site Description, Soil Collection, and Physicochemical Analyses

Soil samples were collected at the Fazenda Nova Vida (10°10′5″S and 62°49′27″W) situated in the western Amazon Basin, state of Rondonia, Brazil, in February of 2004. The annual precipitation reaches 2,200 mm and the annual temperature averages 25.5°C at this site (Bastos and Diniz, 1982), with only two distinct seasons: dry and wet. Soils are classified as Ultisols (US soil taxonomy), representing 22% of the Brazilian Amazon basin (Moraes et al., 1995). This area experiences the highest rate of deforestation in the Amazon basin, driven in large part by conversion of forest into pasture for cattle production.

Three different land use types were selected for sampling: primary forest (hereafter referred to as forest), a pasture established in 1987, and a secondary forest resulting from pasture abandonment in 1994 and subsequent natural re-colonization by forest plants. The common procedure for pasture establishment is aerial seeding of two fast growing grasses *Urochloa brizantha* (Hochst. ex A. Rich; formely the genus *Brachiaria*) and *Panicum maximum* without the use of chemical fertilizers or agricultural machinery. The plant community composition of the secondary forest is a mix of woody species and grasses (Feigl et al., 2006). Twenty-five independent soil samples per treatment (0–10 cm) were collected 50 m apart from each other in order to cover the 3 ha area plots, kept on ice, and transported to an on-site laboratory, where they were randomly combined into groups of five and sieved with a 2 mm mesh. The resulting five replicates per treatment were stored at -80°C prior to molecular analysis (Cenciani et al., 2009).

The soil under all land uses in this study is classified as a red–yellow podzolic latosol (Kandiudult) with sandy loam texture (Feigl et al., 2006). Soil samples were sieved with a 2-mm mesh and used for total C and N determination with an Auto Analyzer LECO TruSpec CN at the Centro de Energia Nuclear na Agricultura, University of Sao Paulo, Brazil. The soil attributes pH, organic matter, base saturation, cation exchange capacity, Al^+^ saturation, C/N, moisture, potential acidity, and elemental analysis of P, S, K^+^, Ca^+2^, Mg^+2^, Al^+3^, H^+^, B, Cu, Fe, Mn, Zn were analyzed at the Department of Soil Sciences, University of Sao Paulo, Brazil, as previously described (Cenciani et al., 2009).

### *Verrucomicrobia* Primer Design and Evaluation

A *Verrucomicrobia*-specific 16S rRNA gene-targeted primer was designed using the ARB software package and the SILVA small subunit reference database that contains 4,781 high quality verrucomicrobial sequences (Prüsse et al., 2007). The target region for the reverse primer VER_673R (5′ TGC TAC ACC GWG AAT TC 3′) was identified between nucleotide locations 673 and 690, according to the *Escherichia coli* numbering system (Gutell, 1993), taking into consideration the presence of a hairpin followed by a non-canonical pairing of six nucleotides in the 16S rRNA gene secondary structure. When combined with a phylum-specific forward primer VER_37F (5′ TGG CGG CGT GGW TAA GA 3′ Buckley and Schmidt, 2001) the amplified region encompasses the hypervariable regions V1–V4, ensuring accurate taxonomic identification. Primer specificity was further tested against 2,765,278 16S rRNA gene aligned sequences using the Check Probe function of the Ribosomal Database Project II^2^ (RDP; Cole et al., 2009) and the Basic Local Alignment Search Tool (BLAST) program from the National Center for Biotechnology Information (NCBI; Altschul et al., 1990). This primer VER_673R yielded a perfect match for 3,211 out of 3,939 high quality verrucomicrobial sequences present in the RDP II, including those belonging to all 133 isolates available throughout the phylum. When searching the database, 35 other sequences were found to be targets for this primer. These sequences, previously identified as *Acidobacteria* (3), *Firmicutes* (4), *Bacteroidetes* (1), *Chloroflexi* (1), *Proteobacteria* (12), and unclassified (14), came from environmental clone libraries and, upon our inspection, could not be classified with at least 80% confidence using the naïve Bayesian rRNA RDP classifier.

The specificity of the *Verrucomicrobia* 16S rRNA gene-targeted primers was experimentally tested with genomic DNA extracted from pure cultures of members of the phylum *Verrucomicrobia* and isolates belonging to the above groups, when available. No PCR amplification was observed for any groups other than *Verrucomicrobia* strains (Supplementary Figure SF1). All isolates yielded positive amplifications with the universal 16S rRNA gene eubacterial primers 8F and 1492R, indicating that the DNA was suitable for amplification.

### DNA Extraction and PCR Amplification

Total genomic DNA from soil (0.25 g) was extracted using the Power Soil DNA MoBio DNA Extraction kit (Mobio Laboratories Inc., Carlsbad, CA, USA). Amplification reactions were performed in a final volume of 25 μl containing 10 ng of DNA template, 0.2 μM of each *Verrucomicrobia* 16S rRNA gene specific primer, 2.0 U of Accuprime *Taq* DNA polymerase (Invitrogen Life Technologies, Carlsbad, CA, USA), and 1X Accuprime PCR buffer II with the following components: 20 mM Tris-HCl (pH 8.4), 200 μM deoxynucleotide triphosphates, 1.5 mM MgCl_2_, and 0.5 mM KCl. Amplification was initiated with a 5-min denaturation step at 95°C, followed by 30 cycles of denaturation at 94°C for 30 s, primer annealing at 55 C for 30 s, extension at 68°C for 1 min, then a final extension for 10 min. Aliquots (5 μl) of the PCR products were visualized on ethidium bromide-stained 1% agarose gels.

### Clone Library Sequencing

Cloning and transformation were carried out according to the instructions provided with the TOPO TA cloning kit (Invitrogen Life Technologies, Carlsbad, CA, USA). *E. coli* transformants were grown on Luria–Bertani medium containing kanamycin (50 μg/ml) and subjected to PCR targeting the cloned insert with primers M13F (5′ GTT GTA AAA CGA CGG CCA GTG 3′) and M13R (5′ CAC ACA GGA AAC AGC TAT G 3′) under the same PCR conditions as above. Amplified products were purified with the kit ExoSAP-IT (USB Corporation, Cleveland, OH, USA) according to manufacturer’s instructions and sequenced on an ABI PRISM 3100 automatic sequencer using Big Dye chemistry (Applied Biosystems, Foster City, CA, USA) at the University of Texas Genomics Core Facility (Arlington, TX, USA). A total of 750 sequences were screened and edited in the software Sequencher v.4.2. (Gene Codes Corporation, Ann Arbor, MI, USA). The chimera-check software from the RDP was used to screen sequences for chimeric origin^3^. Taxonomic classification was carried out using the online RDP classifier function.

### Taxonomic and Phylogenetic Diversity Analyses

Trimmed sequences were aligned with the software MUSCLE (Edgar, 2004) and visually inspected before creating a distance matrix with DNAdist software. Operational taxonomic units (OTUs) were established using the furthest neighbor algorithm implemented in the softwared package Mothur (Schloss et al., 2009) with clustering set at 1% of dissimilarity, in accordance to species definition proposed by Stackebrandt and Ebers (2006). OTU richness and Faith’s phylogenetic distance (Faith, 1992) were calculated with the software picante (Kembel et al., 2010) in the statistical platform R, and diversity estimators (Shannon and reciprocal Simpson 1/D) were calculated with mothur. Both α and β diversity indices were calculated according to the definition of Whittaker (1972). Taxonomic similarity was calculated by using the Bray–Curtis index, while phylogenetic similarity was calculated by using weighted UniFrac (Lozupone et al., 2007). Patterns of community structure were visualized by non-metric multidimensional scaling (NMDS) of taxonomic (Bray–Curtis) and phylogenetic (weighted UniFrac) similarities. Taxonomic and phylogenetic community compositions were compared among sites by using analyses of similarity (ANOSIM; Clarke, 1993). Class level differences were analyzed with the Library Compare function of the RDP using a confidence threshold of 90%.

For phylogenetic measures of diversity, a phylogenetic tree was constructed with MUSCLE-aligned sequences (Edgar, 2004) and a maximum-likelihood tree was constructed using the gamma + I model with the program PhyML (Guindon et al., 2010). Circular trees were constructed and displayed using the Interactive Tree of Life website^4^.

Statistical significances for species richness and diversity indices were calculated with one-way analysis of variance (ANOVA). Fitting of soil variables onto principal coordinate analysis (PCoA) was performed with the vegan package of the software R^5^, and the significance of correlation assessed after 999 random permutations.

### Quantitative Real Time PCR

Triplicate qPCRs for each soil sample were performed in 20 μL volume containing 1X iTaq fast SYBR Green supermix with ROX as internal reference (Bio-Rad, Hercules, CA, USA), 50 nM of each *Verrucomicrobia* specific primer, and 5 ng of total soil DNA. The ABI 7300 real time PCR system (Applied Biosystems, Calrsbad, CA, USA) was used for quantification. Information about amplification conditions, sensitivity of the quantitative PCR assay for *Verrucomicrobia* specific primers, standard curves, and controls can be found in Supplementary Material.

### Nucleotide Sequence Accession Numbers

Partial 16S rRNA gene sequences were deposited in the GenBank database under the accession numbers JF410109 to JF410858.

## Results

### *Verrucomicrobia* Species Richness

Sampling effort was evaluated through rarefaction curves (Supplementary Figure SF2). The number of OTUs did not reach an asymptote for any of the replicates among the three treatments, but consistently lower numbers of OTUs were observed for the primary forest in comparison with the other two land use treatments. A similar pattern was observed when replicates were pooled within each treatment. Although non-parametric indices are prone to underestimate diversity (Bohannan and Hughes, 2003), they are particularly appropriate for comparative studies such as this one using standardized sample sizes.

**FIGURE 1:**
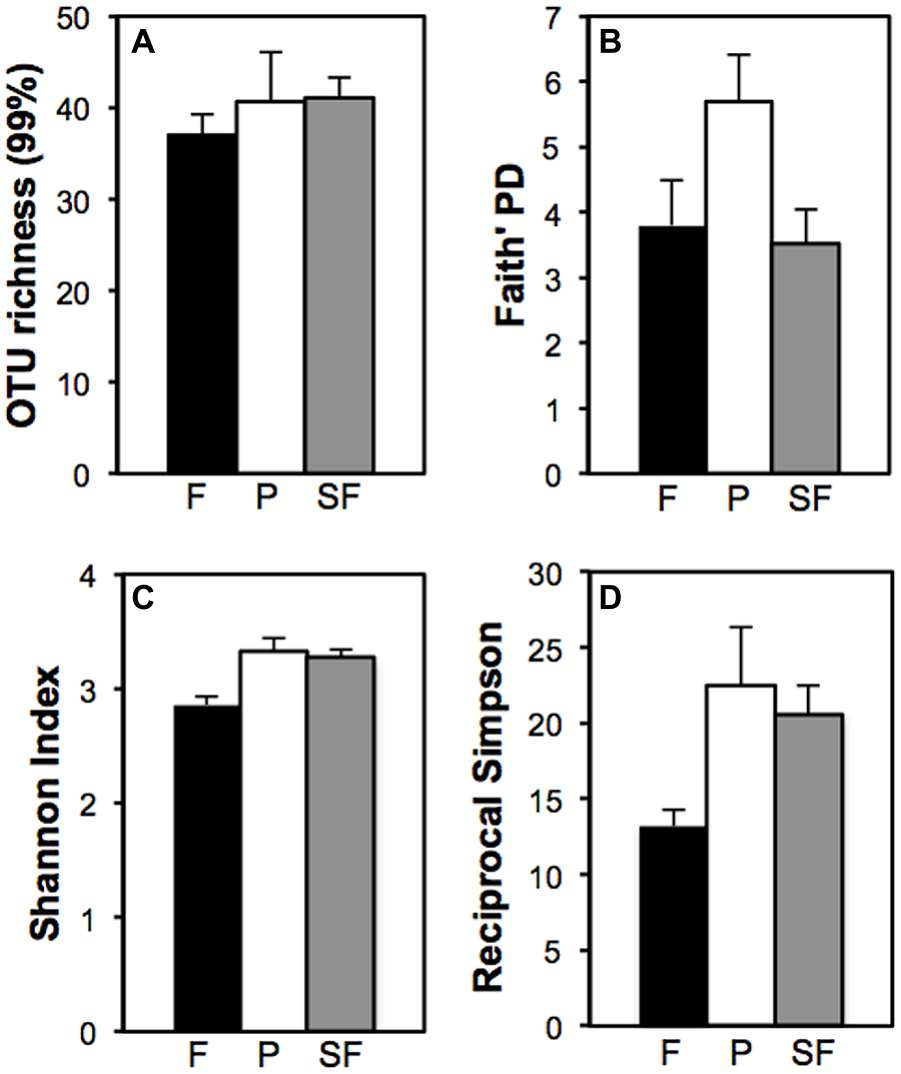
**Land use effect on the local diversity of *Verrucomicrobia* in forest (F, black), pasture (P, white), secondary forest (SF, gray): **(A)** total species richness, **(B)** Faith’s phylogenetic diversity, **(C)** Shannon Index, and **(D)** Reciprocal Simpson Index (1/D).** Bars represent SEs.

Two indices, OTU richness and the Faith’s phylogenetic distance, were used to estimate *Verrucomicrobia* diversity. The number of OTUs did not differ significantly among treatments. While 37 OTUs were observed in the primary forest, plots of pasture and secondary forest had 40 and 41 OTUs, respectively (**Figure 1A** – out of 50 sequences). Faith’s phylogenetic diversity index was significantly higher for the pasture [*F*_(2,12)_ = 3.12, *P* = 0.05] in comparison to primary and secondary forests, which were similar to one another (**Figure 1B**).

### α and β Diversity Analyses

Both non-parametric-derived indices of α diversity, Shannon and the reciprocal of Simpson (1/D), had significantly lower values for the forest than the other two treatments [*F*_(2,12)_ = 8.07, *P* < 0.01; *F*_(2,12)_ = 3.53, *P* = 0.05, respectively] (**Figures 1C,D**). Pasture and secondary forest had similar Shannon indices of 3.33 ± 0.11 and 3.28 ± 0.07, respectively. The reciprocal of the Simpson index was slightly higher for the pasture (22.46 ± 3.93) in comparison to the secondary forest (20.56 ± 1.92).

**FIGURE 2:**
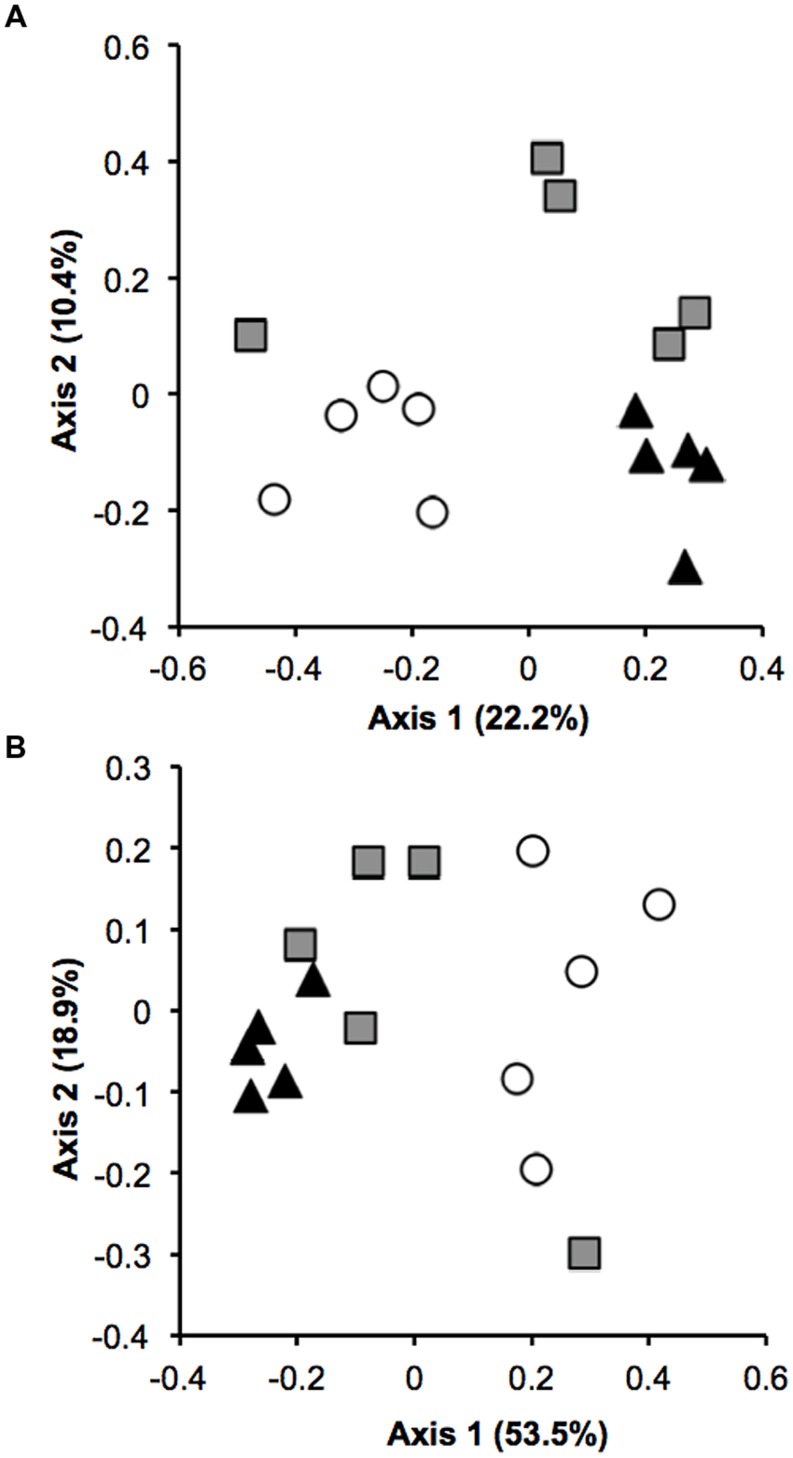
***Verrucomicrobia* community composition of forest (triangle), pasture (circle), and secondary forest (square).** Non-metric multidimensional scaling (NMDS) plot of **(A)** taxonomic similarity (Bray–Curtis) and **(B)** phylogenetic similarity (weighted UniFrac).

The *Verrucomicrobia* community composition from pasture soils was significantly different from that observed in primary and secondary forests. Analyses of similarity using both taxonomic (*R* = 0.501, *P* < 0.001) and phylogenetic (*R* = 0.476, *P* < 0.001) measures revealed a distinct separation between pasture and forest samples (**Figures 2A,B**). Two measures of β diversity were used to estimate the similarity in community structure among soil samples. Both UniFrac (phylogenetic) and Bray–Curtis (taxonomic) coefficients decreased with forest-to-pasture conversion, a trend which was reversed for both indices with pasture abandonment and re-establishment of secondary forest [*F*_(2,57)_ = 5.86, *P* < 0.001; *F*_(2,12)_ = 32.2, *P* < 0.001, respectively, Supplementary Figure SF3].

### Phylogenetic Novelty

**FIGURE 3:**
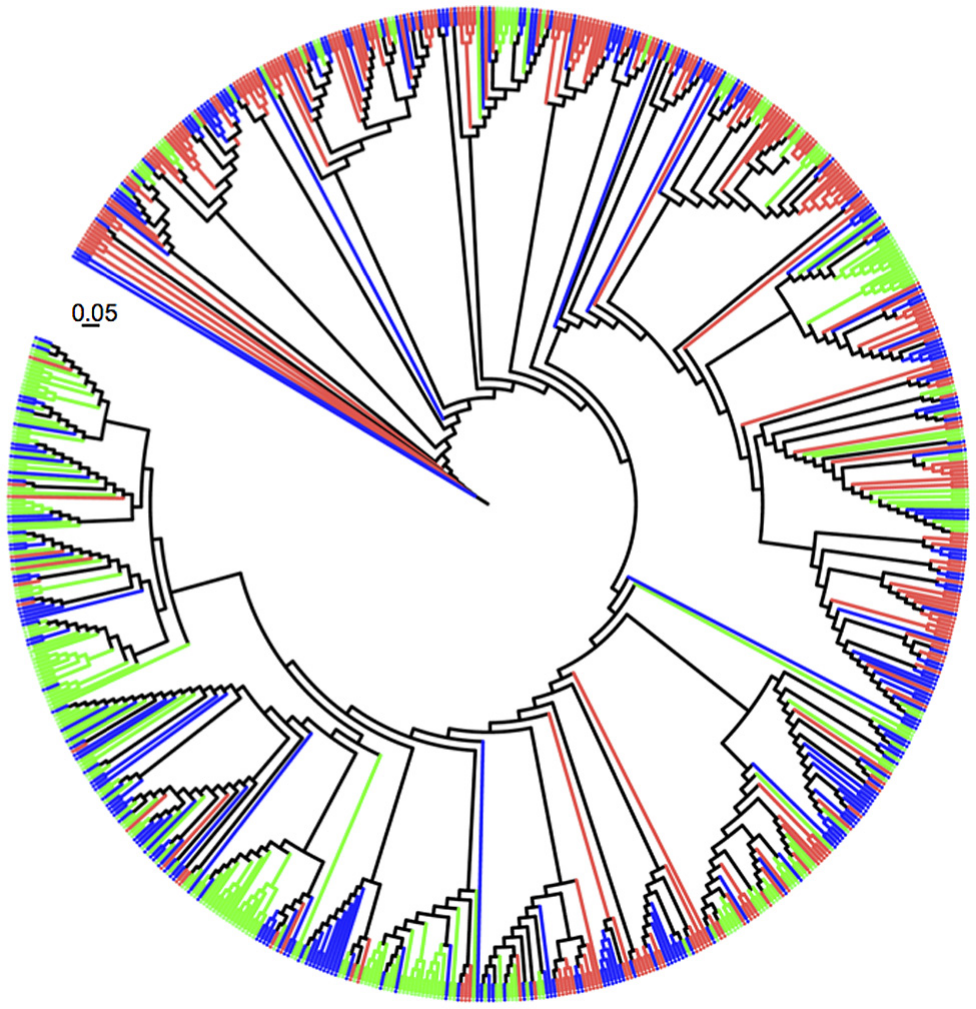
**Unrooted maximum likelihood tree of 750 sequences belonging to *Verrucomicrobia* 16S rRNA genes recovered from soils: Amazon forest (green), pasture (red), and secondary forest (blue).** The scale bar represents the number of substitutions per nucleotide position.

All 750 sequences were identified using the naïve Bayesian rRNA RDP classifier based on 99% sequence identity (**Figure 3**). A large majority of pasture *Verrucomicrobia* sequences grouped into clades different from those obtained from forest soils. *Verrucomicrobia* sequences cloned from the secondary forest samples were evenly distributed throughout the phylogenetic tree. A total of 302 sequences (40.3%) retrieved in this study were not previously observed in any other study. The majority of the novel sequences (*n* = 124) originated from the forest samples, followed by the secondary forest (*n* = 113) and the pasture (*n* = 65).

**FIGURE 4:**
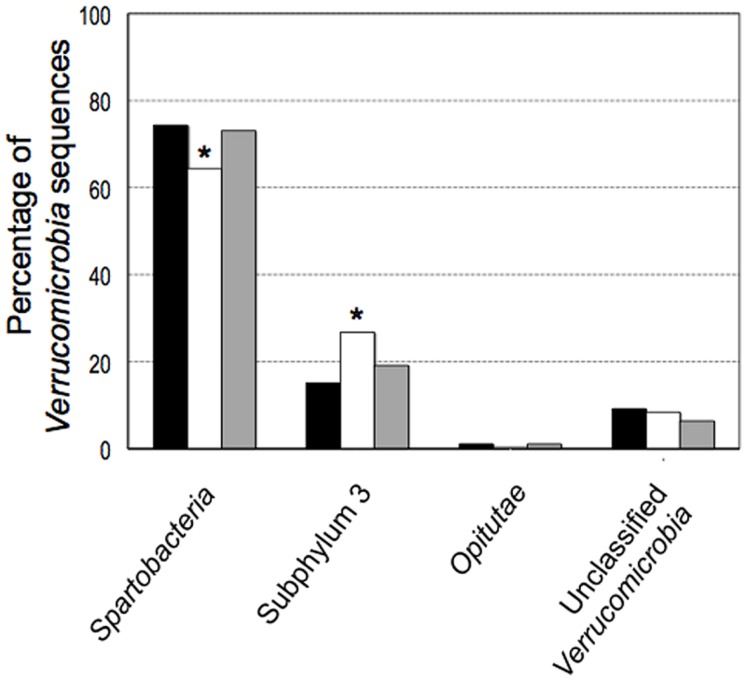
**Phylogenetic comparison among classes of the phylum *Verrucomicrobia* 16S rRNA gene sequences from different land uses in the Amazon: forest (black), pasture (white) and secondary forest (gray).** Symbol ^∗^ indicate significant differences at *P* = 0.01.

The majority of the sequences (90%) belonged to two classes, *Spartobacteria* and subphylum 3. The relative proportion of sequences classified as subphylum 3 in the pasture (26.8%) was significantly higher (*P* < 0.01) than the number of subphylum 3 sequences found in the forest (15.2%, **Figure 4**). There was an equivalent decrease of 11.6% in the number of *Spartobacteria* 16S rRNA gene sequences recovered from pasture samples. The relative proportions of *Verrucomicrobia* sequences in the primary and secondary forests were similar for both the *Spartobacteria* and subphylum 3 with a minor variation of 1.2 and 4%, respectively.

### Influence of Soil Factors on the *Verrucomicrobia* Community

To explore potential drivers of the community, we performed environmental fitting of 22 different soil variables onto the ordination plot via Principle Coordinate Analysis (PCoA, Supplementary Figure SF4). Five variables were linked to *Verrucomicrobia* phylogenetic community structure, with total C yielding the most significant results (*P* < 0.05). Increases in organic matter, total N, and pH were linked to 16S rRNA gene sequences obtained from pasture, while an increase in potential acidity (H + Al) was linked to those from forest samples.

### Abundance of *Verrucomicrobia*

The detection limit was established to be approximately 10^3^ copies of the target sequence per gram of soil with linear standard curves over six orders of magnitude. Gene copy numbers were consistently higher (*P* < 0.05) for pasture samples (1.9 × 10^7^ copies per gram of soil), followed by secondary forest (9.1 × 10^6^) and primary forest soil samples (2.4 × 10^6^).

## Discussion

Soils are known to have higher taxonomic and functional diversity than any other environment (Whitman et al., 1998). Earlier estimates suggested that 3.8 × 10^3^ to 10^7^ microbial species are present in a gram of soil (Curtis et al., 2002; Gans et al., 2005) and 32 phyla have already been detected (Janssen, 2006). We reason that if one is interested in understanding how soil microbial communities respond to environmental disturbances, focusing on particular groups is crucial, since not all groups are likely to respond in the same manner.

To capture the diversity of the phylum *Verrucomicrobia*, we designed a new 16S rRNA gene PCR primer (VER_37F) and paired it with a previously tested primer VER_673R (Buckley and Schmidt, 2001). This new primer pair differs from *Eubacterial* primers (515F/806R) proposed by Bergmann et al. (2011). While the primers 515F/806R are relatively unbiased against the *Verrucomicrobia*, they are not specific for this phylum. The primer pair VER_37F-VER_673R was highly specific for the target phylum when tested *in silico* and experimentally with the recovery of 750 *Verrucomicrobia* sequences from Amazon soils. Although our sample is not as comprehensive as one obtained from a high-throughput sequencing study, the longer reads we generated provide more detailed taxonomic assignments for lower rank hierarchies (Wang et al., 2007) of the numerically dominant OTUs present in our soil samples than would be possible with a high-throughput study. Both methods use PCR based information and large-scale patterns observed in soils with the PCR dependent method T-RFLP (Fierer and Jackson, 2006) were unchanged when the same samples were subjected to pyrosequencing (Lauber et al., 2009).

Our results indicate the presence of a diverse *Verrucomicrobia* community that was not previously observed in tropical rainforests through the use of oligonucleotides targeting the domain *Eubacteria*. An important outcome of our targeted approach is the high number of novel sequences observed. Using an OTU definition based on 99% sequence identity, 40.2% of the *Verrucomicrobia* sequences were novel, suggesting that the microbial diversity of tropical ecosystems remains largely unknown. More importantly, this high diversity in Amazon soils is impacted by forest-to-pasture conversion. Both non-parametric indices (Shannon and the reciprocal of Simpson) showed increased values of community diversity for soils collected from pasture. These findings are in agreement with previous studies, in which forest-to-pasture conversions resulted in increased α diversity of bacterial communities (Jesus et al., 2009; Rodrigues et al., 2013). We also documented an increase in β diversity (Anderson et al., 2011) as measured by a decrease in taxonomic (Bray–Curtis) and phylogenetic (UniFrac) similarities. The response of *Verrucomicrobial* beta diversity contrasts the response of the *Bacteria* as a whole, which showed increased taxonomic homogenization following pasture conversion. This further demonstrates the utility of focused analysis on particular microbial groups, which can often differ from patterns observed within the larger community.

It is possible that the differences we observed across land type could be the result of spatial variability in soil conditions that were present in the three sites before land use change occurred. This is unlikely, for two reasons. First, all of the study sites have identical soil type, drainage, slope, and are geographically located close to each other (Cenciani et al., 2009). Second, this chronosequence of land uses has been systematically studied over the past 25 years and spatial variability was found to be small in comparison to land use history (Moraes et al., 1995; Steudler et al., 1996; Neill et al., 1997).

We observed that the community composition of *Verrucomicrobia* was altered with land use change in two distinct ways. First, the relative proportions of taxa varied with land type. There is a clear distinction between clades formed by OTUs observed in the forest and those found in the pasture (**Figure 3**, note that forest sequences marked in green are mostly at the bottom of the circular tree while pasture sequences, in red, are positioned at the top). This alteration can be attributed to the increase in 16S rRNA gene sequences belonging to the subphylum 3 and a concurrent decrease in the number of *Spartobacteria* sequences. Out of the seven taxonomic classes within the *Verrucomicrobia* (Hedlund, 2011), these two groups comprise the majority of the 16S rRNA gene sequences and isolates retrieved from soil (Joseph et al., 2003; Sangwan et al., 2004; Janssen, 2006). We cannot characterize the increase in the relative proportion of subphylum 3 sequences as a shift in dominance, as the *Spartobacteria* sequences remain above 60% of the total in all sites. However, our results are consistent with habitat filtering, the process by which habitat characteristics exert selection pressure on community composition (Webb et al., 2002). We documented that at least 11.6% of the verrucomicrobial community was altered as a consequence of conversion. It is currently unknown if these forest inhabitants carry unique traits. Genomes of the first isolates of both *Spartobacteria* and subphylum 3 have been sequenced (Kant et al., 2011a,b), and a comparative analysis may provide insights into their ecophysiological differences and reasons for habitat selection. Secondly, the abundance of *Verrucomicrobia* increased with conversion of forest to pasture. We hypothesize that this response is a direct result of rich exudates released by the large root system of C4 plants, such as grasses (Feigl et al., 1995; Haichar et al., 2014). Two lines of evidence support this is hypothesis: (A) higher abundances members of the *Verrucomicrobia* have been observed in the plant rhizosphere in comparison to bulk soil (Idris et al., 2004; Kielak et al., 2008; Jesus et al., 2010; Rocha et al., 2010); and (B) attempts to isolate members of *Spartobacteria* from soil have only been successful when plant polymers or sugars were added to the medium (Sangwan et al., 2004). Taken together, the above results suggest that plants may be an important driving force, albeit difficult to quantify, on the community composition of *Verrucomicrobia* in soils.

Given the genetic and ecological consequences of tropical biodiversity loss, we asked whether there are signs of resilience within the forest *Verrucomicrobia* community (Allison and Martiny, 2008). Our results indicate that restoration of community composition is under way after 7 years of disturbance (pasture from 1987 to 1994), followed by 10 years of reestablishment of a secondary forest (from 1994 to 2004). After many years of cessation of the disturbance, the above ground community has not returned to its original species composition (Feigl et al., 2006), nor have many of the soil attributes been restored to their original values (Cenciani et al., 2009). Pairwise comparisons of the secondary forest samples revealed that the *Verrucomicrobia* community structures are similar to those observed for the primary forest. Furthermore, *Verrucomicrobia* communities of primary and secondary forests respond to the same environmental variables (Supplementary Figure SF4). Because phylogenetic relationships are not always predictors of microbial physiology, it remains to be determined whether similar phylogenetic taxa perform similar functional roles in the restored ecosystem. Nonetheless, our results are encouraging from the standpoint of community conservation and restoration ecology. Approximately 50% of abandoned pastures in the Amazon rainforest are estimated to be in a secondary forest succession (Davidson et al., 2012). Young tropical forests are increasing in importance for genetic diversity conservation and stability of degraded environments, but their microbial communities, nutrient cycling processes, and ecosystem services remain understudied. Our study, although limited, suggests that membership recovery may be possible.

**FIGURE 5:**
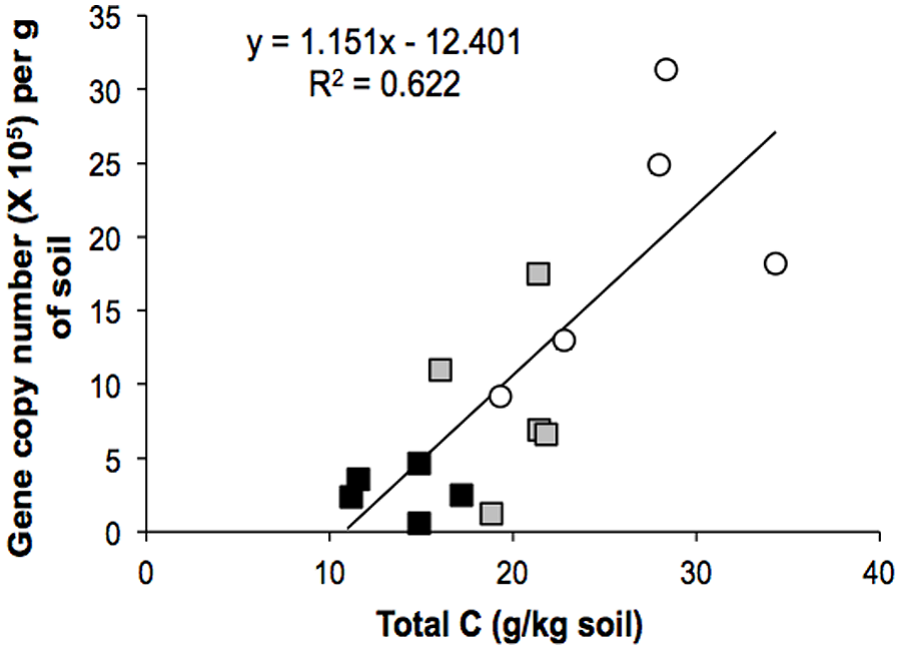
**Regression analysis between the abundance of *Verrucomicrobia* 16S rRNA genes and total carbon in the Amazon soils.** The correlation coefficient (*r*) between the two variables is 0.80 (*P* = 0.0016). Symbols: forest (black square), pasture (open circle), secondary forest (gray square).

Environmental biotic and abiotic characteristics are important determinants of the ecological niche occupied by a particular population and we sought to correlate the presence of members of the *Verrucomicrobia* with 22 soil variables. While potential acidity (H + Al) was the only variable linked to the *Verrucomicrobia* community from the primary forest, increases in organic matter, pH, total *N*, and total *C* were significantly associated with those from the pasture. These soil properties are long lasting effects of the slash and burn procedure (Neill et al., 1997) and drastic alteration of plant species composition to actively growing grasses, with high deposition of *C* to soil (Feigl et al., 1995). We examined the influence of *C* content on the abundance of *Verrucomicrobia* and observed a strong correlation (*r* = 0.80, *P* = 0.0016; **Figure 5**), indicating that *C* may be an important factor in determining the structure of this particular microbial group. Carbon resource heterogeneity has been suggested as a driving force for microbial community structure (Zhou et al., 2002) and it is likely that changes in the above ground community will alter carbon content and availability of different carbon-containing compounds. Our results suggest that the *Verrucomicrobia* community increases its abundance in soil in response to *C*, in agreement with previous metagenomic studies that found that the distribution of carbohydrate metabolism genes from *Verrucomicrobia* were associated with shifts in *C* dynamics (Fierer et al., 2013).

## Conclusion

Although land use change has profound consequences for plant and animal biodiversity, its effect on microorganisms is not well understood. By targeting the phylum *Verrucomicrobia* at class level of taxonomic and phylogenetic resolution, we documented changes in the relative abundance of specific groups with forest-to-pasture conversion, as well as an increase in both alpha and beta diversity. We also identified carbon content as an important environmental factor associated with soil *Verrucomicrobia* abundance. By solely focusing on the *Verrucomicrobia*, we were able to (1) increase the number of novel *Verrucomicrobia* 16S rRNA gene sequences detected, (2) characterize changes in taxonomic and phylogenetic diversity at the class level, and (3) make inferences about the environmental requirements for yet-to-be cultured members of this phylum. Although we documented strong shifts in response to deforestation and pasture establishment, 10 years after disturbance, the *Verrucomicrobia* community shows signs of recovery to its original composition, suggesting that this group shows resilience to ecosystem modifications.

## Conflict of Interest Statement

The authors declare that the research was conducted in the absence of any commercial or financial relationships that could be construed as a potential conflict of interest.

## Footnotes

1 http://rdp.cme.msu.edu

2 http://rdp.cme.msu.edu/index.jsp

3 http://rdp8.cme.msu.edu/cgis/chimera.cgi.

4 http://itol.embl.de.

5 http://www.r-project.org
