# Supplementary material for: Forest-to-pasture conversion increases the diversity of the phylum Verrucomicrobia in Amazon rainforest soils
Source: Front Microbiol. 2015 Jul 30;6:779. doi: 10.3389/fmicb.2015.00779 (PMC4519759; doi:10.3389/fmicb.2015.00779)
Supplement: Supplementary file 1 [file Data_Sheet_1.PDF]

SUPPLEMENTARY INFORMATION

Real Time PCR standard curve. The DNA template for standard curve development was obtained from a randomly selected environmental clone. The *Verrucomicrobium* 16S rRNA gene insert was amplified with M13F and M13R primers, followed by purification with Qiaquick PCR purification kit (Qiagen Inc., Valencia, CA) and quantification in the Nanodrop. The gene copy number was calculated according to the formula: Copy number = (A × B)/MW, where “A” is the DNA concentration, “B” is the Avogadro’s constant (6.02×10^{23} expressed as number of genes per mol) and “MW” is the molecular weight of the insert. Tenfold serial dilutions ranging from 10^2 to 10^8 gene copies per reaction were used to construct a standard curve ($y = - 4.89x + 52.95$). The reactions were subjected to a denaturation step of 3 min at 95°C, followed by 40 amplification cycles of 30 s at 95°C, 30 s at 50°C, and 1 min at 68°C. Additionally, a melting curve from each individual reaction was run to confirm amplification specificity. The threshold cycle values (Ct) were plotted against the log of the gene copy number and the line slope was obtained by regression analysis. There was a linear relationship between the log of the target DNA copy number and the calculated threshold cycle value across the specified concentration range ($r^2 = 0.994$-0.998). The amplification efficiency was calculated according to the formula $Ae = 10^{(-1/slope)}$ and varied between 1.58 and 1.61. The detection limit was 10^3 copies. Control reactions with no template were undetectable.
