# Supplementary figures and images for: Forest-to-pasture conversion increases the diversity of the phylum Verrucomicrobia in Amazon rainforest soils
Source: Front Microbiol. 2015 Jul 30;6:779. doi: 10.3389/fmicb.2015.00779 (PMC4519759; doi:10.3389/fmicb.2015.00779)

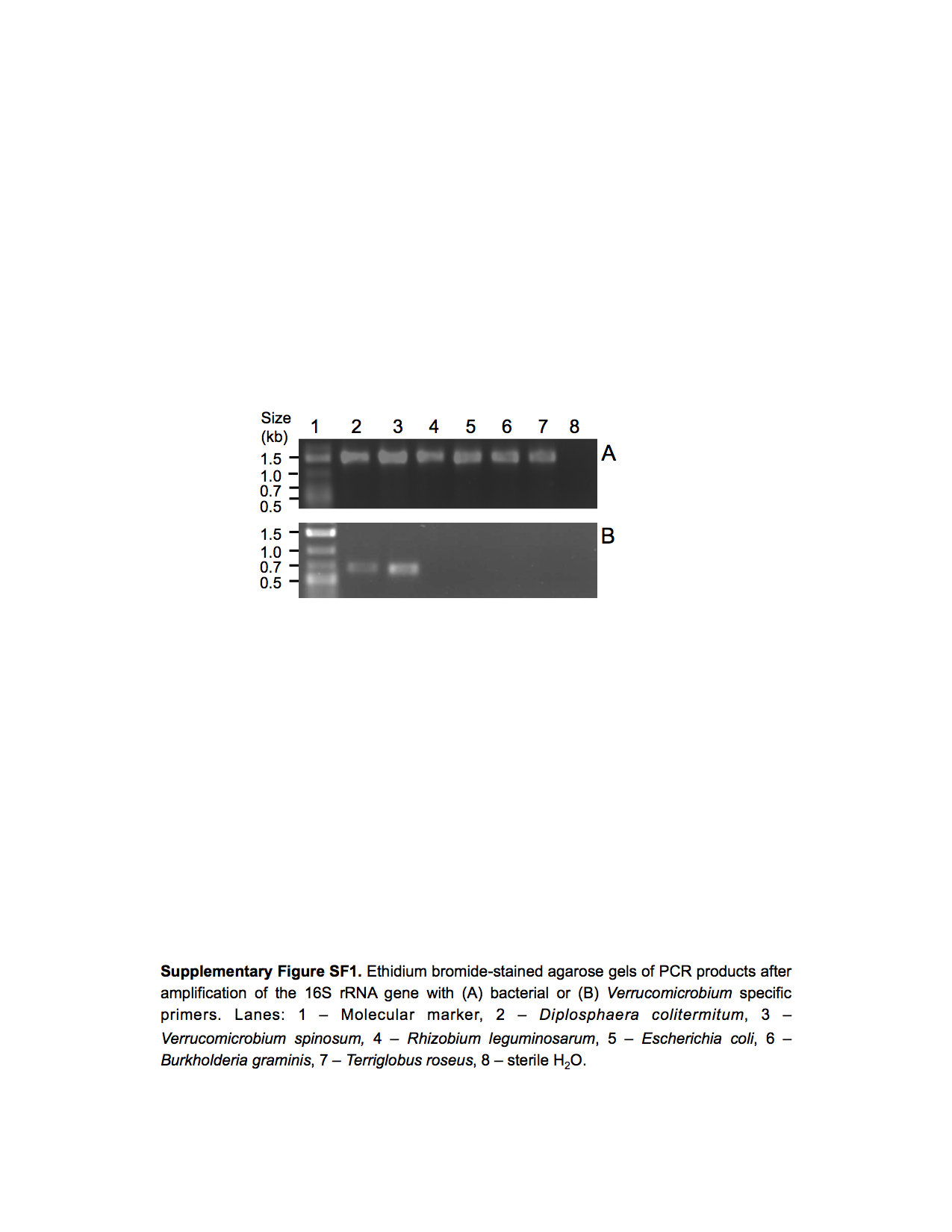

Supplement: Supplementary file 2 [file Image_1.TIFF]

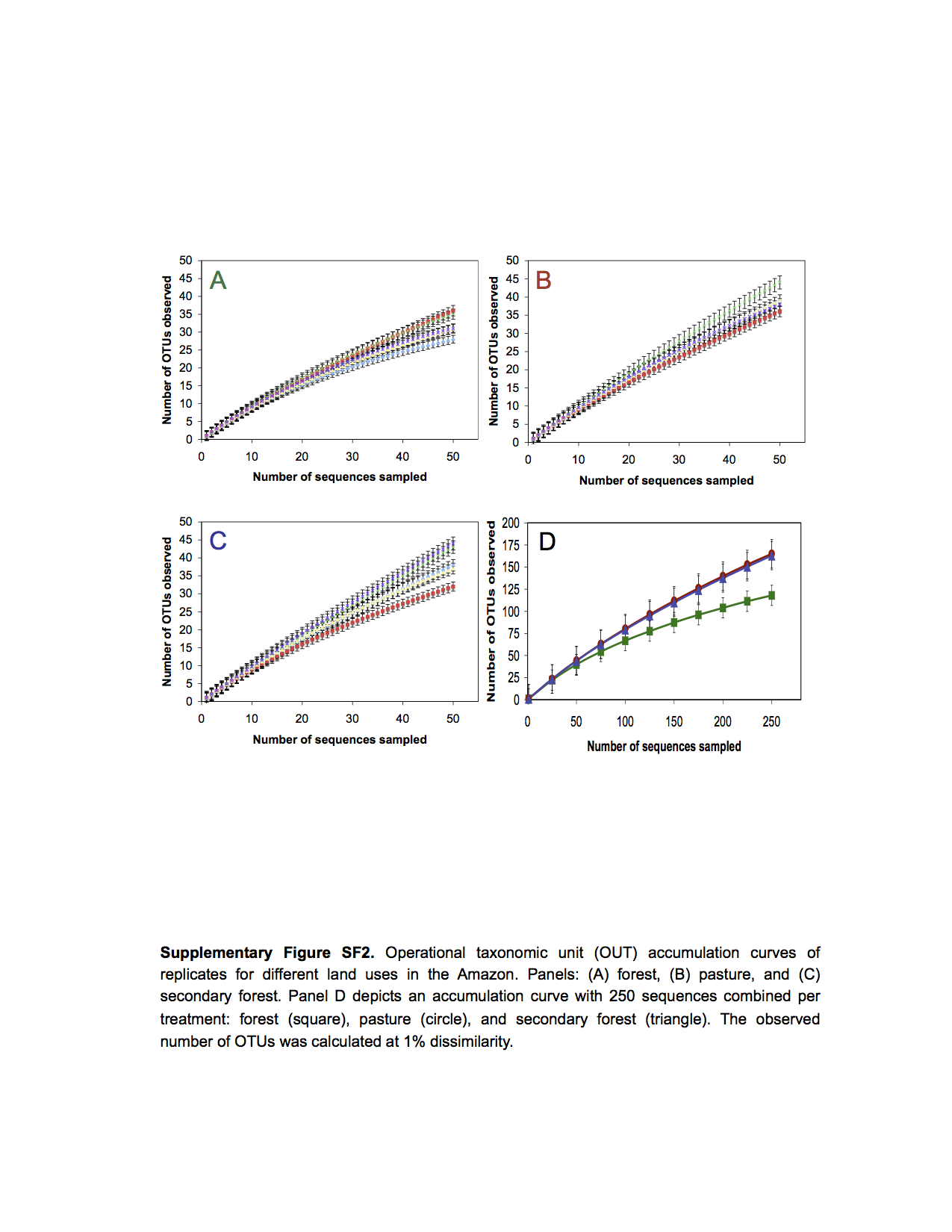

Supplement: Supplementary file 3 [file Image_2.TIFF]

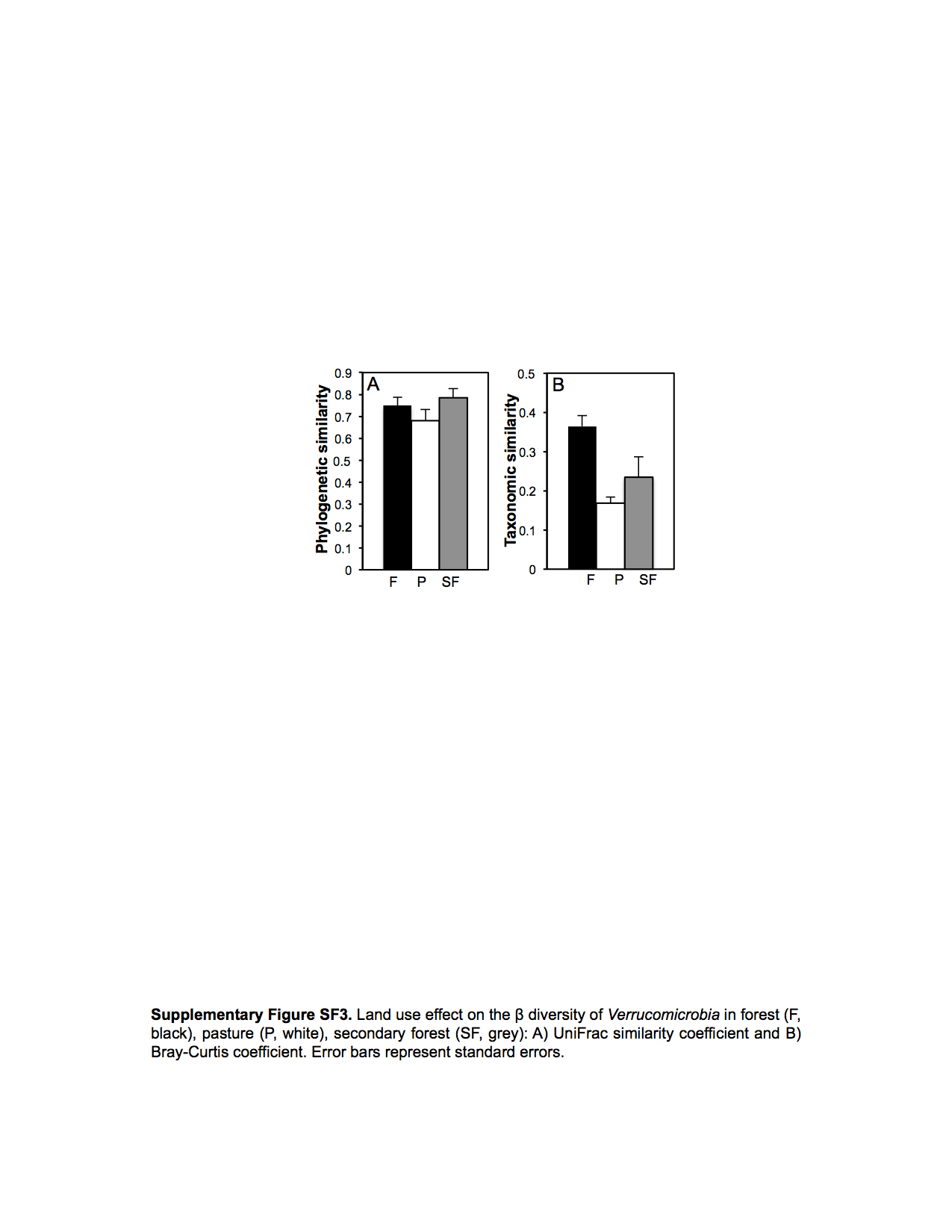

Supplement: Supplementary file 4 [file Image_3.TIFF]

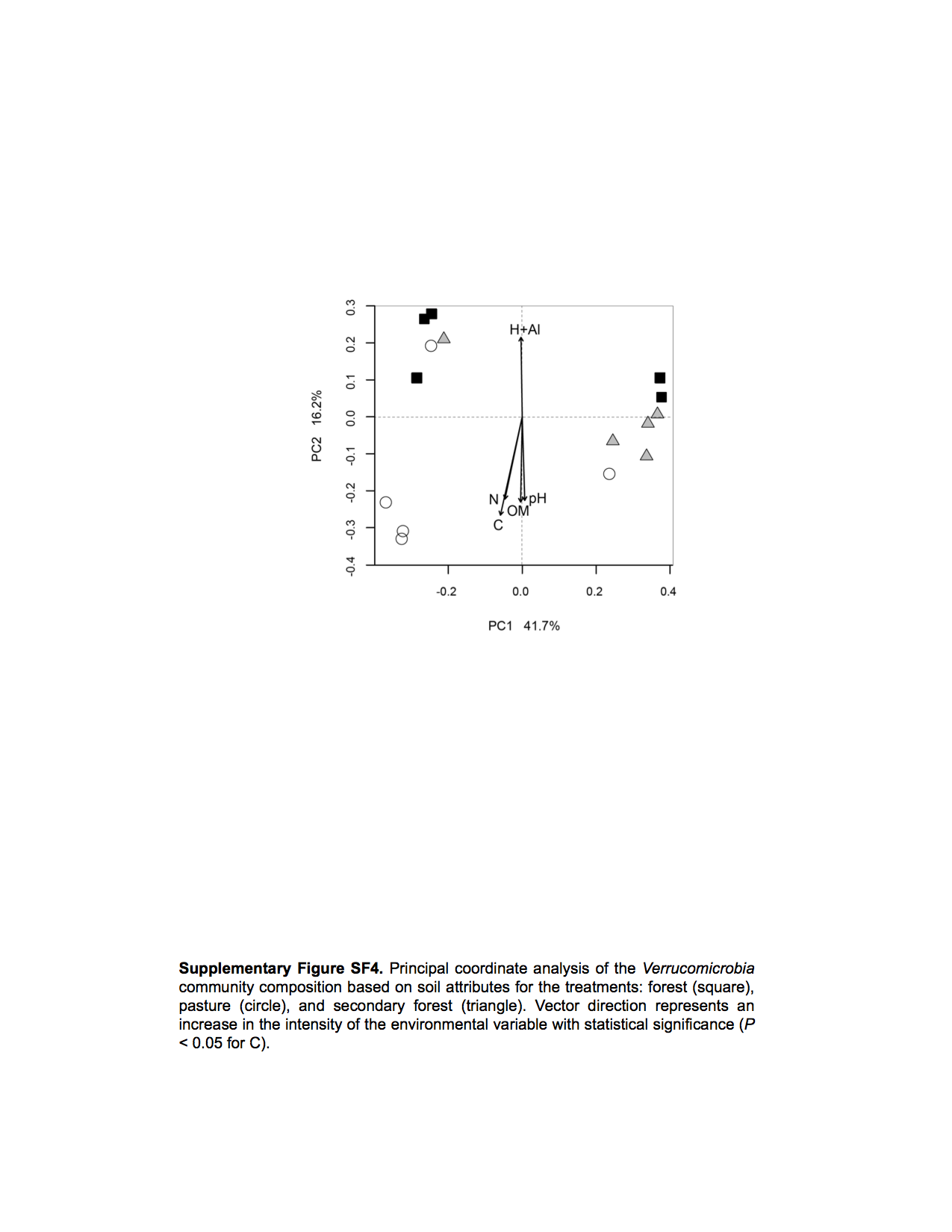

Supplement: Supplementary file 5 [file Image_4.TIFF]
